# Proposal Writing Training and Idea Development for Early‐Career Researchers Based on Constructive Alignment, Co‐Creation and Active Learning Strategies

**DOI:** 10.1002/ece3.72162

**Published:** 2025-10-27

**Authors:** Friederike Hoffmann, Mahaut de Vareilles, Linling Chen, Catherine Downy, Nadine Goris

**Affiliations:** ^1^ Geophysical Institute University of Bergen Bergen Norway; ^2^ Department of Earth Sciences University of Bergen Bergen Norway; ^3^ Nansen Environmental and Remote Sensing Center Bergen Norway; ^4^ NORCE Climate and Environment Bergen Norway; ^5^ Bjerknes Centre for Climate Research Bergen Norway

## Abstract

In today's research landscape, which is ever more dependent on external funding, early career researchers (ECRs) urgently need competence in idea development and proposal writing. However, generalized lectures provided by many higher education and research institutions lack practical elements such as hands‐on skill training and support measures to coach the ECRs through the process of developing their own proposal. This limited support often leads to proposals of low quality, low success rates, and low motivation to engage further in proposal writing. To move away from this “learning‐by‐failure,” we developed a novel concept for training in proposal writing, constructively aligning learning outcomes with students' needs, co‐creating course content with teachers and students, and using active learning strategies. The main novelty of this concept lies in students iteratively and interactively developing their own research ideas into project proposals while learning how to write proposals. Over the past 10 years, we have successfully run this concept as a 2‐day workshop and as a 5‐month class for ECRs in climate sciences.

Most of the proposals developed during our courses were submitted, and the application success rate of 15%–30% is well above the average for the targeted funding schemes. Participants whose proposals were rejected nevertheless appreciate the high learning outcome and the peer support and are motivated to revise and re‐submit their proposals. We see that increasing numbers of our ECRs are eager to develop and submit their own research ideas, which, considering the high success rates, brings both scientific and economic benefits to our institutions. We hope other research and higher education institutions will adopt our course concept, allowing more ECRs to benefit from co‐created proposal writing training which directly aligns learning outcomes with students' immediate needs.

## Graphical abstract

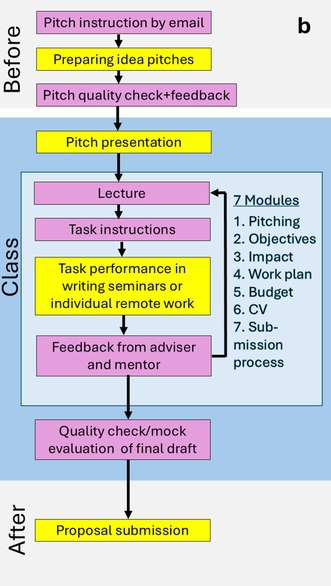

## 1 Introduction: Early Career Researchers (ECR) Need Better Training in Idea Development and Proposal Writing

In today's research landscape, which is getting ever more dependent on external funding, competence in targeted idea development and proposal writing becomes a key transferable skill for early career researchers (ECR). We know that many research and education institutions are aware of the needs of the community, and thus offer grant writing seminars through research schools, career centers, or as part of the curriculum. While providing essential generalized information and recommendations, the offers we are aware of rarely include practical elements, such as hands‐on skill training on how to develop a research idea, which concrete steps to take for turning this idea into a project description, as well as systematic coaching through the process of developing their own project proposals. Individual scientific mentors and supervisors often provide invaluable support with a focus on scientific quality control of the proposals, but few research institutions have a standardized and formalized process to secure individual coaching for all their ECRs. ECRs often face significant challenges in securing funding (de Winde et al. 2021; Andrews et al. 2020; Pannell et al. 2019). The experience of failure and being left alone in a complex and confusing process leads to low motivation of ECRs to engage further in proposal writing, or even to continue at all with their scientific career (Sithole et al. 2017).

## 2 A Novel Training Concept

To move away from this practice of “learning by failure” and demonstrate that first proposal submissions can be successful, we developed a novel training concept for proposal writing, based on concepts that have been demonstrated to positively influence learning. These concepts are constructive alignment of the learning outcomes with the students' needs (Biggs 1999, 2003), co‐creation of the course content and knowledge by teachers and students together (Bovill 2020) and several active learning strategies (Freeman et al. 2014) as outlined below. During the past 10 years, we have performed proposal writing training annually at the Bjerknes Centre for Climate Research in Bergen, Norway, and continuously co‐developed, together with the students, our own course concepts that we present here.

### 2.1 Institutional and Strategic Context

Our courses are open for all ECR who have a connection to the Bjerknes Centre for Climate Research, a collaborative umbrella organization of which the authors' home institutions are members. Our definition of ECR includes doctoral candidates, post‐doctoral fellows, and researchers within about 8 years of having obtained their doctoral degree. This matches the eligibility criteria for the main ECR‐targeted funding instruments of the European Research Council (ERC), the European Commission's Marie Sklodowska Curie Program (MSCA) and the Research Council of Norway (RCN) that our course participants are heading for. Organizers, teachers, and mentors (see Section 2.3) perform these course‐related tasks as in‐kind contributions, which are approved by our home institutions. Furthermore, our courses are embedded in research schools and training programs of our home institutions. The Bjerknes Centre for Climate Research and all its member institutions consider this effort as one of our measures to reach our strategic goals for supporting Early Career Scientists in line with European policy such as the European Charter for Researchers, to which we are committed.

### 2.2 Two Course Formats: Workshop and Class

**FIGURE 1:**
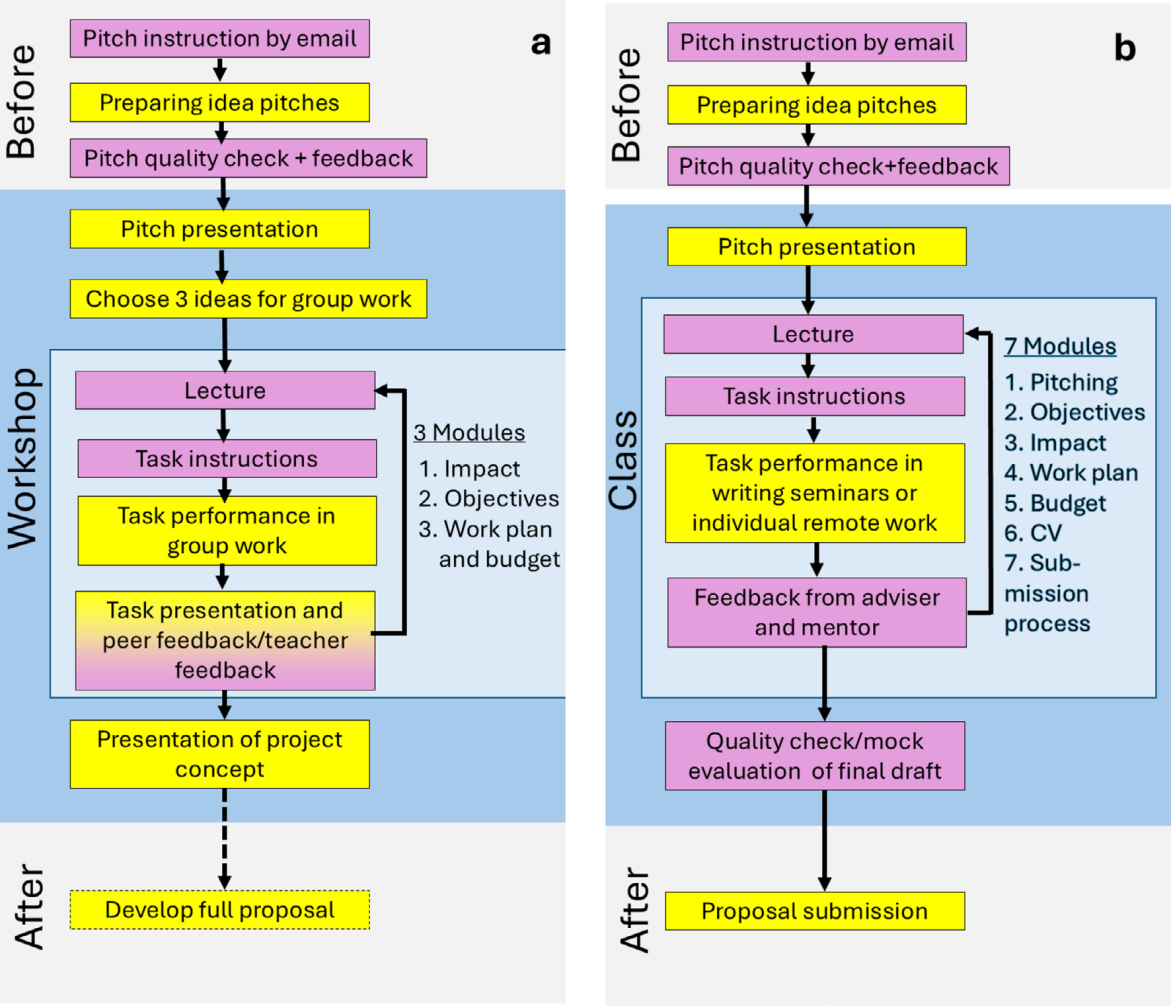
Course concept of proposal writing training: (a) Format 1 “The Workshop”: The 2‐day workshop to develop project concepts and (b) Format 2 “The Class”: The weekly class to develop full proposals. Yellow: student tasks; purple: teacher tasks.

We offer this new training concept in two course formats, Format 1: the Workshop and Format 2: the Class, as presented in Table 1 and Figure 1a,b. Both formats target ECRs with little or no experience in proposal writing and build competence to plan, write, and submit a successful project proposal.

**TABLE 1:** Comparison of key characteristics of the two course formats of the proposal writing training: The 2‐day workshop for project concept development (Format 1), and the weekly class for full proposal development (Format 2).

	Course Format 1 (workshop)	Course Format 2 (class)
Venue	On‐site workshop	Online lectures + on‐site writing seminars, weekly alternating
No. of participants	Max 15	Unlimited (lectures), max 20 (seminars)
Duration	2 full days	1.5 h/week, 8 lectures, 7 writing seminars, spread over 5 months
Main result	Co‐developed project concepts, based on the project ideas of 3 participants	Competitive proposals developed individually by each participant based on their own project ideas
Learning outcome—competence	Competence to plan, structure, write and submit a successful project proposal	
Learning outcome—skills	Idea pitching, project concept development, structure a project description, draft a project budget	
		Scientific CV writing, alignment with targeted call for proposals
Learning outcome—knowledge	Funding opportunities, funding mechanisms	Call requirements, evaluation processes, evaluation criteria
Target group participants	Early career researchers in climate sciences with little or no experience in scientific proposal writing	
	Both with and without a concrete project idea	With a concrete project idea, and a plan to submit a proposal within the coming year

To give a comprehensive idea of how these concepts are incorporated into our courses, an example of the program of the 2023/2024 class and the 2024 workshop is shared as Appendix S1.

The main difference between the two formats is their concrete output. In course Format 1 (the Workshop), the research ideas of three of the participants are co‐developed by three working groups into three project concepts. We limit the number of participants to 15. As all groups present their work and give feedback during the workshop, it is our belief that allowing for more than three groups is too time‐consuming and that more than five participants per group limits our goal of active learning. In course Format 2 (the Class), where we do not limit the number of participants, all participants develop their own project ideas into a competitive and quality‐controlled research proposal that is ready to be submitted for funding. Consequently, the Class targets ECRs with their own project ideas, while the Workshop is also open for ECRs who do not yet have a clear project idea. However, the Workshop can only be performed if at least three of the participants bring project ideas suitable for development into project concepts during the workshop. The workshop's success therefore depends on having enough participants with suitable project ideas. We solve this issue by prioritizing participants with their own project ideas when assigning slots for the (always over‐booked) workshop, and by providing constructive feedback to the project pitches that the students send to us before the workshop starts. Our experience (based on 10 given workshops from 2015 to 2024) is that approximately half of the 15 admitted participants bring a project idea, and that most of these ideas are suitable to be developed into project concepts during the group work. Building the workshop around the participants' own project ideas and including principles of co‐creation and constructive alignment makes the course content authentic and relevant to the students.

The two courses can be taken individually or in a sequence, first the Workshop and then the Class. Participants who did not get their project idea chosen for group work in the Workshop can develop it during the Class instead, and those who developed their project concept during the Workshop can build on this proposal concept during the Class. Even though there is some overlap in the teaching content between the two course formats, this is not considered a drawback by the students since the focus of both course formats is on skill development, concrete conceptual planning, and proposal writing. The Class provides more in‐depth information so that the different course formats meet the same students at different stages of their skill development. Many students experience good learning outcomes by only taking the Workshop, and students only taking the Class still manage to develop a competitive project proposal during the Class. See Section 4 for feedback on the different course formats by our students.

### 2.3 Roles of Teachers and Mentors

Research advisers of the different member institutions at the Bjerknes Center for Climate Research act *as teachers and organizers* of both course formats: they develop the teaching material and concept through co‐creation with the students (see Sections 3.6 and 4), present the lectures, and supervise group work and writing seminars. Working daily with pre‐ and post‐award support for grants, all advisers have a background in different fields of climate or biological sciences and experience with applying for and managing their own research projects. Some are expert evaluators and review panel members for the European Commission and other national and international research funders. In addition to teaching, the advisers provide *individual coaching* to the participants in between the Class modules or after the Workshop, give feedback on individual remote work, or support the development of the project concept into a full proposal, respectively. They also perform a mock evaluation of the final proposal, with a focus on non‐scientific evaluation criteria (see Section 3.5).

When developing a full proposal during the Class, participants are encouraged to identify a *scientific mentor*. The scientific mentor's main task is to inspect the scientific content of the project proposal. Based on the project pitch (see Section 3.2), they “stress test” the proposed research idea early in the process of project development: they evaluate if the project idea is ground‐breaking, ambitious, and feasible; and suggest modifications if necessary. Furthermore, they may perform a mock evaluation of the final proposal a few weeks before the submission deadline, with a special focus on evaluation criteria related to scientific excellence (see Section 3.5).

## 3 Key Elements of Our Novel Training Concept

### 3.1 Concrete and Tangible Output: Project Concept and Full Proposal

Both courses lead to concrete outputs:

- In the Workshop, the research ideas that are co‐developed into three project concepts contain the basic elements of a project description, that is, objectives, work plan, draft budget, main deliverables, and expected impacts (see Section 3.3). They represent a hands‐on experience of how to develop a project concept for all participants. For those participants whose research ideas were selected for development, the project concepts provide a good basis to develop a full project description. This development can be done individually by the student in collaboration with their supervisor and research adviser, or in the Class, which typically starts a few months after the Workshop.
- In the Class, each participant brings their own project idea and finishes the Class with a competitive and quality‐controlled research proposal which is ready to be submitted for funding.

These concrete and usable products come in addition to the general learning outcomes and competence building (see Table 1) and represent the main difference between our course concept and the other commonly available training offers in proposal writing that we are aware of.

### 3.2 Idea Pitching and Quality Check

Both course formats start with idea pitching (Porter 2011). The participants receive guidelines a few weeks before the Class/Workshop starts and send their pitches to the teachers for quality control and feedback. A document containing pitching instructions for students and a teacher's assessment template for the feedback is shared as Appendix S2. Quality‐controlled pitches are then presented by the students in the first session of the Class/Workshop. For the Workshop, only those who plan to bring a project idea receive the guidelines. The research ideas expressed in the pitches then provide the basis for the selection of the three ideas for development (based on popular vote) and for all further group work tasks (see Figure 1a; Section 2.1).

For the Class, all participants get feedback on their pitches from peers and teachers during the class and improve/sharpen their pitch accordingly during individual remote work. Here, the peers are instructed to focus their feedback less on the scientific content but more on the quality of the pitch. The students are encouraged to use their optimized pitches to approach senior scientists in their field, for example their scientific mentor (see Section 2.3) who then provides a scientific quality control (“stress test”) of the proposed project idea. Students are also encouraged to present their pitch in research group meetings or similar for more peer feedback. Based on all feedback, they may modify their idea and move on with their proposal—or decide that the idea is not (yet) ready to be developed into a full proposal. Taking this go/no‐go decision at this early stage of proposal development saves these students a lot of time and frustration and ensures that the proposal has good potential for success. For ideas that pass the “stress test,” the quality‐controlled and updated project pitch can be further used to approach potential collaboration partners and stakeholders, and to quickly inform institution leaders, colleagues, etc. about the planned initiative.

We consider idea pitching an essential skill in any job situation where one's own idea or plan needs to be convincingly communicated to a team or superior. We are therefore already teaching idea pitching in our introductory class for Master students (course GEOF‐301 in the curriculum of University of Bergen).

### 3.3 Project Concept Development

#### 3.3.1 From “Why” to “What”—Plan the Proposal From the End

Strong research proposals start by explaining what you will achieve when the project is successful, which problems you will have solved, and why this matters in a broader context. Planning and communicating a research proposal from the end (“why”) makes a strong and convincing proposal (Porter 2011). However, our experience shows that most researchers develop and present their proposals from the start: this is what we know—and this is what we want to do (“what”). Explaining the need for the proposed research first gives reviewers valuable context and stimulates their interest to dig into the technical details (Porter 2011).

An important learning outcome of our pitching exercises is thus to understand the need to define a problem and its importance in a broader context before planning a solution. The “problem” section of the pitch is what our students struggle the most with. Once this is defined, the project idea often gets much more focused and convincing.

In a similar way, we train the students to define what they want to achieve (objectives) before planning what to do (work plan). Many researchers, even seniors, phrase project objectives as a list of tasks, rather than as measurable and targeted goals. This weakens proposals, since the reviewers may get an idea of what will be done in the project, but not why. We teach our students to develop SMART (specific, measurable, achievable, realistic, targeted) objectives, including clear metrics for success, before planning concrete work tasks.

Our courses therefore always start with planning the “why” (impact, objectives) before planning the “what” (work plan, budget, partners). This is reflected in the order of the teaching modules (see Figure 1; Appendix S1).

Additionally, we have found that starting with defining the objectives rather than the work tasks leads to greater flexibility and openness to the project approach and the planned methods. This often leads to a more targeted approach and a more careful choice of methods.

#### 3.3.2 Science Is Complex, Writing Is Linear

We encourage our students to plan the proposal following the order of our course modules, rather than following the order of a given project description template. Yet, all these proposal aspects are linked, and during a creative proposal development process, the scientist will have to work on all these aspects in parallel and iteratively. We therefore frequently encourage the students, in both course formats, to re‐visit their outputs from the previous course modules and consider if revision is needed in light of their recent developments. An important learning outcome of our courses is to understand and handle the challenge of translating a complex scientific idea into a linear text.

### 3.4 Learning Cycles With Different Active Learning Methods and Tools

In both course formats, the content of the different modules is communicated through learning cycles (Kolb 1984; Vince 1998).

For the Workshop, a learning cycle consists of a lecture, the task instruction for the group work, the task performance by the groups, and the task presentation followed by feedback from peers and teachers in a plenary session (see Figure 1a). Groups of five participants, which include the owner of the project idea, are created for each of the three chosen project ideas. The cycles start with an *introductory lecture* by one of the teachers, to communicate the background knowledge needed to fulfill the group work task. The lecture finishes with presenting the *group work instructions*. These instructions, as well as templates where the groups fill in the results of their work, are available on a shared online workspace (shown in Appendix S3). This tool makes the group work more efficient and presentation in plenary technically easier.

Another measure to make the group work more efficient and ensure active participation of all students is to allocate specific roles among the group members (e.g., Møgelvang and Nyléhn 2023): focus keeper, note keeper, presenter. These roles are rotated among the five group members in the four group work sessions. We introduced this measure as the idea owner is naturally most eager to make the group work a success. In previous years, we experienced that this person could dominate the group work, while other group members became more passive and disengaged. Now we see that the allocation of concrete roles activates all group members. Even though the idea owner is often still the driving force in the group work, it is our experience that the entire group gets enthusiastic as the project idea develops.

When group work starts, each group is accompanied by one of the teachers to answer practical questions and to make sure that the group manages to organize its work. The teacher can also moderate the discussion when necessary. When the group work goes well, the teacher will leave the group alone but follow the progress of the tasks on the shared online workspace.

Each group then presents the results of their group work in a plenary session where the presenter shows the group's results on the shared workspace and receives peer feedback. The other group members have 4 min to discuss and reflect on the presentations. They get feedback guidelines (Appendix S3), in which they should agree on two positive aspects and one suggestion for improvement, which they then share in plenary. Finally, the teachers give additional feedback to quality control the feedback process and to assess if the group work results show that the learning outcome for this session was achieved.

The modules of the Class (Figure 1b) consist of online lectures, which also include a presentation of the task instructions. We chose the online format to give greater flexibility to the students. The students have 2 weeks to perform the work tasks before the next module starts. The tasks can be performed individually and remotely, or during the on‐site writing seminars, which take place in the weeks between the lectures. There is always at least one teacher present during the writing seminars to answer questions and to support the students with performing their tasks, if necessary. Not all students make use of the writing seminars, but those who do report them as very helpful for their progress (see Section 4). Each student/project proposal is supervised by one of the teachers, who provides feedback on the remote tasks performed and coaches the student throughout the proposal development process. The scientific mentors provide feedback concerning the scientific aspects of the proposal (see Section 2.3).

For both course formats, all teaching material—lecture slides, work instructions, and templates, examples of successful proposals—are shared with the students and can be looked up both during and after the workshop/class.

### 3.5 Mock Evaluation of the Final Proposals

For the Class, teachers and scientific mentors offer a mock evaluation of the final proposals, 2 weeks before the submission deadline at the latest. The evaluation templates of the envisaged funding scheme and project type are used to identify strengths and shortcomings for each of the evaluation criteria. Scientific mentors are asked to focus on the criteria related to scientific excellence and methodology, while the course teachers/research advisers focus on structure, readability, consistency, clarity, and completeness of the information provided, in relation to non‐scientific evaluation criteria, particularly those targeting the project's impact and implementation. This represents the final quality check of the proposals. By doing this, we make sure that our ECRs will send in proposals that are considered to be of high quality, both scientifically and how they are presented.

### 3.6 Interview With Successful Applicants

A highlight of the Class is a special seminar where we interview two successful grant applicants to talk about their proposal writing experiences. This special seminar is a result of co‐creation, having been originally suggested and co‐designed with students from previous classes. For the interview, we would choose ECRs from the Bjerknes Centre community—preferably those who joined one of our courses in previous years. Our experience is that it encourages our students a lot to see that “one of them” has just been successful. We also ask these applicants to share experiences about any unsuccessful proposals they may have submitted, and we see that learning about past failures from others reassures and builds confidence for the course participants.

We announce this event to the entire community of our research center and particularly encourage senior scientists to join and also share their experience. In addition to our Class participants, several other early‐career scientists usually join the seminar and contribute actively by asking questions or joining the discussion.

## 4 Success Rates and Participants' Feedback

We can only provide success rates for proposals from the Class format, as it is only the goal of the Class (and not the Workshop) to leave the course with a competitive and quality‐controlled research proposal ready to be submitted for funding (see Section 3.1). We track the outcome of the participants' submissions as part of our regular jobs as research advisers, where we follow up and monitor the success of all project proposals sent from our departments. Of the 15–18 participants that typically followed the class to the end in the past years, we see that a majority (ca. 10–15 participants) submitted a proposal within 12 months after the end of the class. We further see that typically 2–3 of these got funded in the same year, or a year later after having been submitted a second time. This gives a success rate of 15%–30% for our class participants, which is higher than average success rates for the targeted funding schemes (e.g., 14% for ERC Starting Grant, ERC 2024, and 15% for the Early Career Grant of the Research Council of Norway 2025). Furthermore, the non‐funded proposals often received above average grades, and we see that these candidates are often very successful in obtaining external funding in the following years. This is consistent with recent findings that individuals with “near misses” (submitting excellent proposals which fall just below the funding threshold) systematically outperform candidates with “narrow wins” in the longer run (Wang et al. 2019)—if they continue with their scientific career despite this early career setback. Even participants whose proposals are rejected with a low score are motivated to revise and re‐submit their proposal or to try again with another project idea. These students sometimes choose to take the Class another time, as it helps them to stay on track.

**FIGURE 2:**
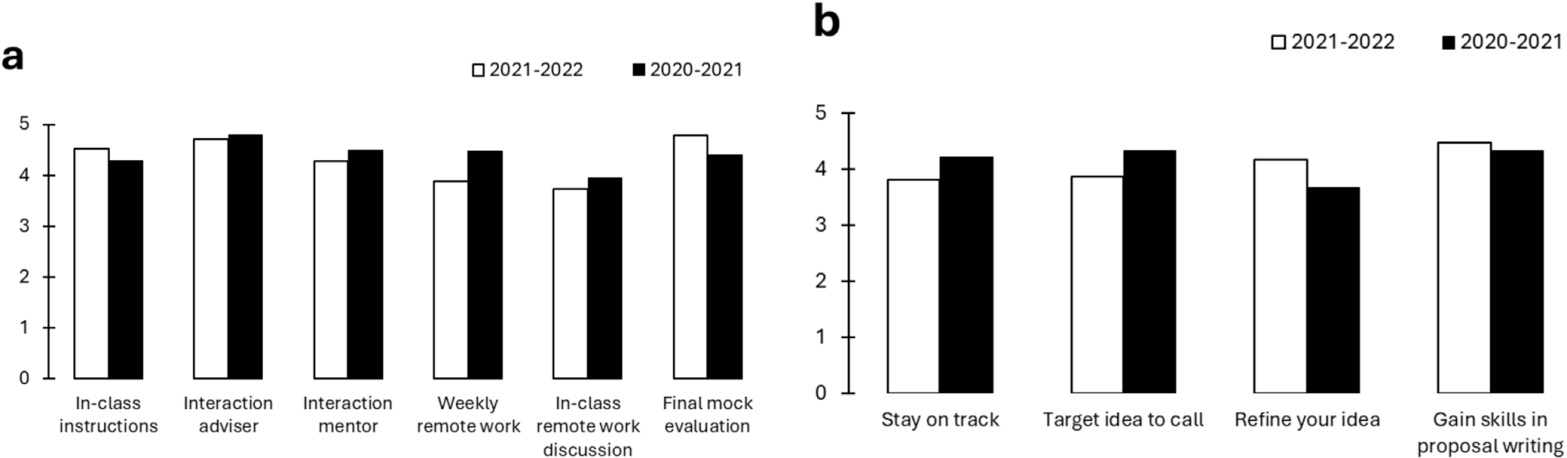
Evaluation of course Format 2, the weekly class to develop full proposals. Anonymous evaluation by the participants in the years 2021/2022 and 2020/2021. Average values, scale 1–5 where 5 is “most useful”. Number of participants that answered the survey = 17 (2021–2022) and 18 (2020–2021). (a) Participants answered the following questions: How useful were the following measures? (1) In‐class instructions for proposal writing, including in‐class interaction with advisers, (2) individual interaction with the research adviser outside of classes, (3) interaction with the scientific mentor, (4) receiving and performing weekly remote work tasks, (5) in‐class discussion of remote work, and (6) final mock evaluation of the proposal. (b) Participants answered the following questions: Was the class useful for… (1) staying on track and writing the proposal in a timely way? (2) targeting your proposal idea towards call text and assessment criteria? (3) better focusing/refining your research idea? (4) Gaining new knowledge and skills for proposal writing?

Participant feedback for both course formats is gathered each year via anonymous online surveys. Figure 2 shows an example of the participants' feedback for the Class in 2020/2021 and 2021/2022. The participants were asked to rate different elements of the Class on a scale from 1 to 5 where 5 is the highest/most useful. In‐class instructions, direct interaction with the research adviser outside classes, and direct interaction with a scientific mentor received the highest scores, as well as a final mock evaluation of the complete proposal before submission. Weekly remote work to be performed individually in between classes was considered helpful by those who managed to allocate sufficient time to perform these tasks (which not all participants manage). Brief in‐class discussion of remote work was considered less useful by several participants, and they suggested seminars to perform and discuss the remote work instead. Because of this feedback, we introduced in 2023 the current class format of bi‐weekly writing seminars in between the lectures, which allows the participants to perform the remote tasks at their own pace and during a defined time slot, with research advisers present to guide and answer questions individually. Many participants appreciated this offer and joined the seminars to perform and discuss their remote work. This is another example of how the course concept is continuously co‐created.

In line with the objectives of the Class, participants reported that the Class helped them to stay on track with their proposal, to refine their research idea, and (for thematic calls) to better target their idea to the call topic (Figure 2b). Additionally, participants reported that they gained increased skills in proposal writing. This shows that our approach based on constructive alignment of students' needs with the course outputs (finalized proposals) is a powerful tool for teaching proposal writing skills—while, at the same time, coaching the ECRs when writing their first competitive research proposal.

Feedback for the Workshop was collected in the same way, by anonymous online surveys where several workshop aspects were rated on a scale of 1–5 where 5 was highest/most positive. In line with the workshop objectives, the participants reported a high learning outcome in proposal writing and a clear understanding of how the learning outcomes fit into their career, and all the participants would recommend the Workshop to peers (see Table 2). Free text feedback included comments such as “The workshop was very helpful to take my idea from a very nascent stage to give it a basic skeleton;” “what could have taken me months was done in 2 days;” “I have heard, tried out and learned a lot;” “Perfect for a beginner;” and “Thanks for a great class in line with my needs”.

**TABLE 2:** Evaluation of course Format 1, the 2‐day workshop for project concept development.

	2019	2021	2022	2023	2024
I feel that I learned something which is useful to me	4.85	5.00	4.73	4.92	4.81
I have a clear understanding of how the course fits into my research/career	4.77	4.92	4.67	4.83	4.88
The course was what I expected, based on the stated aims and objectives	4.77	4.85	4.67	4.58	4.69
The quality of practical arrangements was good	4.54	4.69	4.43	4.67	4.38
I would recommend the course to other students	5.00	5.00	4.93	4.91	4.62
Overall rating of the course is good	4.85	5.00	4.87	4.92	4.81

*Note:* Anonymous evaluation by the participants in the years 2019 and 2021 to 2024. Average values, scale 1–5 where 5 is most positive. Number of participants = 15.

Another measure of the quality of our courses is that they are very popular among our ECRs. The Workshop, limited to 15 participants for practical reasons, is always overbooked. The Class, with no limitation for participant numbers, attracts 20–25 participants each year, of which 15–18 typically follow the class to the end. Those who drop off will often complete it the following year. We see that nearly all the Post Docs and many of the PhD students of the Bjerknes Centre for Climate Research have taken at least one of our courses during the past years.

In addition to the invaluable learning outcome, the development of excellent research ideas, and the high proposal submission and success rates, we consider the main benefit of both course formats that the students enjoy them. In the free‐text feedback, we frequently receive comments such as: “The class was super,” “I really enjoyed the workshop,” and “Thanks for a great, informative course.” During the Class, we see that the participants find comfort in being with peers who are in the same situation. During the Workshop, we see that the groups can get very eager and engaged as the project concepts develop, and that they especially enjoy opportunities to express their creativity, for example when jointly developing an acronym or a logo for the proposed project. Proposal writing, which often is experienced as a painful and lonely exercise even by experienced scientists, becomes a fun community effort for the ECRs in our courses.

## 5 Recommendations for Other Higher Education Institutions

The course concepts that we have co‐developed with our students over the past 10 years are very successful and relatively easy to implement. We share our concepts here in the hope that others will use them. If implementing our courses in the suggested formats is not feasible, then as a minimum offer, we recommend providing a space for regular writing seminars organized by the students, supervised by a senior scientist or research adviser. For institutions which already offer courses to teach essential generalized information and recommendations on proposal writing, the co‐created seminar format will add aspects of active learning and constructive alignment and will help the ECRs to stay on track when applying the obtained knowledge to their own project proposals.

At our institutions, we see a change in culture since we started to offer these regular courses. We see that more and more of our ECRs are eager to develop their own research ideas and to submit their own research proposals, and that they start developing their own proposals earlier, even during the last years of their PhD. These courses are therefore not only relevant career‐building measures for the ECRs, but useful for our institutions as well, as they benefit both scientifically and economically from the high proposal development activity and success rates of our ECRs.

We hope that our course concepts will be used by many other higher education institutions, so more ECRs in the future can benefit from co‐created proposal writing training which directly aligns learning outcomes with students' immediate needs.

## Author Contributions

**Friederike Hoffmann:** conceptualization (equal), investigation (equal), methodology (equal), project administration (lead), resources (equal), validation (equal), visualization (equal), writing – original draft (lead), writing – review and editing (equal). **Mahaut de Vareilles:** conceptualization (equal), investigation (equal), methodology (equal), project administration (equal), resources (equal), validation (equal), visualization (equal), writing – original draft (equal), writing – review and editing (equal). **Linling Chen:** conceptualization (equal), investigation (supporting), methodology (equal), project administration (supporting), resources (equal), validation (equal), visualization (equal), writing – original draft (equal), writing – review and editing (equal). **Catherine Downy:** conceptualization (supporting), methodology (equal), project administration (supporting), resources (equal), validation (equal), visualization (equal), writing – original draft (supporting), writing – review and editing (equal). **Nadine Goris:** conceptualization (supporting), methodology (equal), project administration (supporting), resources (equal), validation (equal), visualization (equal), writing – original draft (supporting), writing – review and editing (equal).

## Conflicts of Interest

The authors declare no conflicts of interest.

## Supporting information


**Appendix S1:** Examples for Programs of Workshop and Class.

**Appendix S2:** Guidelines to Prepare a Pitch Talk of Your Project Idea.

**Appendix S3:** Bjerknes workshop: Writing successful project proposals—2024.

## Data Availability

There is no research data directly associated with this article. Most of the course information and teaching material is shared as Appendices S1, S2 and S3. Slides of the lectures and raw data of the course evaluations are available from the corresponding author upon reasonable request.
