# Supplementary material for: Proposal Writing Training and Idea Development for Early‐Career Researchers Based on Constructive Alignment, Co‐Creation and Active Learning Strategies
Source: Ecol Evol. 2025 Oct 27;15(10):e72162. doi: 10.1002/ece3.72162 (PMC12559024; doi:10.1002/ece3.72162)
Supplement: Supplementary file 1 — Appendix S1: Examples for Programs of Workshop and Class. [file ECE3-15-e72162-s003.docx]

**Appendix 1: Examples for programs of Workshop and Class**

**Format 1: Bjerknes Proposal Writing Workshop 2024**

“Writing successful project proposals”

From idea to project: Preparing a draft proposal

Course leaders:

Friederike U. Hoffmann, Research Adviser and EU expert evaluator, GFI, UiB

Nadine Goris, Researcher and Research Adviser, NORCE Climate

Mahaut de Vareilles, Research Adviser and Manager of BCPU, GFI, UiB

When:

09. April  9:00 – 16:00

10 April  9:00 – 15:30

Where:

Address of venue

Teaching material:

 Presentations will be shared a few days before the workshop

Group work:

- Bring a laptop (we need at least one per group)
- Find the material for group work here, check if the links work for you

Group work instructions: link

Writing platform: link

**Workshop schedule**

**Day 1:**

From 8:45 Arrival and coffee

INTRO

9:00

Plenary:

Introduction to the workshop

*Goals and structure*

09:30

Plenary:

Presentation of project ideas

*Participants present their project ideas.*

*Oral presentation/interview, 3+2 minutes each*

Explanation of the voting process

10:30 – 11:15 buffer time/break/voting

11:15

Plenary:

Where to submit your proposal?

*Presentation of most relevant funding programs at RCN and EU*

11:30

Plenary:

Election of 3 project ideas.

*Decision on which ideas should be developed to proposal drafts during the workshop. Distribution of participants in 3 groups*

11:45 Lunch break

Block 1 IMPACT

12:30

Plenary:

Lecture Block 1: Impact

*30 min incl. questions*

13:00

*Participants move into break-out rooms.*

Group work 1: Expected results and Impact

*1 hour*

14:00

Plenary:

Presentation of results from group work 1 and peer feedback

*10-15 minutes per group*

14:30 – 14:45 Coffee break

Block 2 OBJECTIVES

14:45

Plenary:

Lecture Block 2: Objectives

*30 min incl. questions*

15:15

Group work 2: Objectives

+ inclusion of changes according to plenary feedback on group work 1

*45 min*

*16:00 Finish Day 1.*

**Day 2:**

From 08:45 Arrival and coffee

09:00

Plenary:

Presentation of results from group work 2 and peer feedback

*15 min per group + feedback*

09:45

Block 3 WORK PLAN

Plenary:

Lecture Block 3: Work plan and budget

*45min*

10:30 – 10:45 break

10:45

Group work 3: Work plan, milestones, deliverables, budget

+inclusion of changes according to plenary feedback on previous group work

*1 hour*

11:45 LUNCH BREAK

12:30

Plenary: Presentation of results from group work 3 and peer feedback

*15 min per group*

Block 4 SYNTHESIS

13:15

Plenary:

Lecture Block 4 Synthesis

*How to put everything into a project description*

*15 min*

13:30

Group work 4: synthesis of draft proposal

+ inclusion of changes according to plenary feedback on previous group work

*30 min*

14:00 – 14:15 Break

14:15

Plenary:

Presentation of complete draft proposal including changes (10 min pr group)

Feedback from the groups: Are coordinator+group members happy with the result? Did the idea augment during the workshop? Did you learn something?

*30 min*

FINAL

14:45

Plenary

Wrap up, Course feedback, evaluation, certificates

*30-45 min*

*Course finishes at 15:30*

**Format 2: Proposal Writing Class 2024-25**

**Tuesday 10:30 – 12:00, starts 22. October**

**Lectures online on zoom. Link to all the lectures: link**

**Link to the seminar with successful applicants, date, link**

**Writing seminars on-site: Meeting room details**

**Class format and time effort:**

Weekly alternating lectures and writing seminars

- Lecture-sessions consist of 1 hour lecture + up to 30 minutes question/discussion online. Background and guidelines for a specific proposal aspect, instructions for specific remote work on your proposal;
- Writing seminars consist of 1 hour writing on-site: perform remote work, present results and get feedback.

Remote work:

- 1-2 hours/week remote work on your proposal (in addition to writing seminars)
- To be performed by the next class
- Individual meetings with assigned adviser and/or scientific mentor on demand
- You will get individual written feedback on your remote work from your assigned adviser
- Imperfect and tentative individual work is better than no remote work!

**Class schedule**

**30 Sept - Registration Deadline. Instructions for pitch**

All registered participants receive guidelines by mail on how to prepare a pitch of their proposal idea, to be presented in Class 1.

(Autumn holidays 7.- 11. Oct)

**22 Oct – Class 1 – Pitch**

Welcome

Presentation of proposal ideas – class participants.

Presentation of pitches in small groups, peer feedback.

Short info on budget planning, employment requirements (UiB)

Remote work:

- improve your pitch

- identify a scientific mentor (adviser can help you)

- check if your idea is feasible in terms of economy and employment rules

- discuss your idea with your mentor, and/or with your research group

**29 Oct – Seminar with successful applicants - How to succeed with your proposal**

Successful applicants from Bjerknes Centre share their experience

Link to the special seminar, 29th October 2024.

**05 Nov – Class 2 – Objectives, Call requirements, evaluation criteria**

Instructions on how to write project objectives

Information on evaluation criteria and general requirements and formalities.

Remote work:

- formulate project objectives

- get familiar with the call text and the evaluation criteria

- check if your idea addresses tentative call text and all evaluation criteria

- get formal approval for the proposal by your institute

- contact your project economist and start drafting the budget

**12 Nov – Writing seminar**

**19 Nov – Class 3 – Impact**

Instructions how to formulate the impacts and draft a dissemination and communication plan. Guidelines for finding a title and acronym.

Remote work:

- Fill the impact/dissemination table
- Start thinking about title and acronym

**Milestone – 26 Nov**

- **Your project idea is framed, and quality controlled by your mentor**
- **You have the approval of your institute leader to go on with your proposal**
- **The available budget (roughly) covers the planned project costs**

**26 Nov – Writing seminar**

**03 Dec – Class 4 – Work plan and budget 1**

Instruction how to develop your work plan aligned with the budget and the concept of your project.

Remote work:

- define WPs with tasks and associated person months + extra costs
- visualise project concept in a flow chart
- make a GANTT-chart

**10 Dec – Writing seminar**

**17 Dec – Class 5 – Work plan and budget 2 – Nadine**

Instructions how to formulate deliverables and milestones and plan the details of your budget. How to create a risk assessment for your project and include data management.

Remote work:

- define deliverables and milestones; potentially add them to your GANTT-chart
- work on the details of your budget
- meet your economist to update the budget
- Identify the risk associated with your work plan
- Identify needs for data management

**2025**

(06 –09 Jan Bjerknes Retreat)

**07 Jan – Writing seminar**

**14 Jan – Writing seminar**

Work on your project description, advisers will be present for coaching

**21 Jan – Class 6 – CV**

Instruction how to write a CV for specific funding schemes, complementary to respective sections in project description

Remote work:

- Draft your CV
- draft complementary text about you and your project team in the respective chapter (e.g. chapter 3.1 in NFR application)

**28 Jan – Writing seminar – CV check**

Pair-share of your CV, quality check by advisers

**04 Feb - Class 7 – Project description template, online form**

Presentation of NFR project description template. Guidelines how the different proposal components developed in this class will form a project description.

Presentation of NFR online form template, complementarity to the project description. Remote work:

- open and fill in NFR online form, give access to adviser, mentor and economist.
- Check for call updates
- Start writing your project description

**11 Feb Writing seminar – complete your project description**

**18 Feb Writing seminar – complete your project description**

**2 weeks before submission deadline- Final deadline to deliver project description for mock evaluation to your advisor**
