# Supplementary material for: Proposal Writing Training and Idea Development for Early‐Career Researchers Based on Constructive Alignment, Co‐Creation and Active Learning Strategies
Source: Ecol Evol. 2025 Oct 27;15(10):e72162. doi: 10.1002/ece3.72162 (PMC12559024; doi:10.1002/ece3.72162)
Supplement: Supplementary file 2 — Appendix S2: Guidelines to Prepare a Pitch Talk of Your Project Idea. [file ECE3-15-e72162-s001.docx]

**Guidelines to prepare a pitch talk of your project idea**

Name:

Institute:

Targeted call:

Tentative title/topic of your proposal:

| *Instructions for drafting the pitch*  *Answer all questions,* ***max 2 short sentences per question!***  *Entire talk/text:* ***max 3 minutes / max 500 words***  ***No slides! Pitch talk only***  *When presenting: make a short (few seconds) break after each section (I problem, II solution, III vision) so the audience can assimilate and reflect.* |
| --- |

**I – Problem**

Which problem(s) will be solved by the outcomes of your project?

Why is it important to solve this problem (timeliness and relevance)?

What is known/tried so far to solve this problem, why did it not succeed? (very brief!!)

**II - Solution**

What are you planning to do for solving this problem? (very brief!!!)

What is new in your approach?

Why and how do you think it will work (appropriateness and credibility)?

**III - Vision**

What are the immediate benefits of the solved problem?

To what extent will your project address important scientific and societal challenges, directly or indirectly?**Assessment grid and feedback to the student**

Dear [student],

**Brief feedback on your pitch:** 
 
Target audience: [unspecified/unclear/clear] 
 
Purpose of communication: [unspecified/unclear/clear]. 
 
Length: [good/could be longer/could be shorter]

Structure: [say how well it follows the sales pitch structure. Point out only improvements that are necessary to make the group work smoother and that are relatively quick to implement.]

Language: [comment how well the language will be understood by the target audience]

Give a general motivating comment (e.g. “Nice topic !” or “You’re almost there!”)

The pitch is fit for group work! *(if nothing needs to be changed before group work*)

Great if you can provide missing info/correct these minor details before the group work (*if things need to be fixed before group work*)

**Pitch presentation in class**

 Participant reads/presents text:

- Strict total presentation time: 3 min (incl. the seconds for silent reflection).
- One research adviser keeps the time. Be strict and stop presenter, even when not finished!
- Another research adviser makes notes, use pitch guideline, one for each presenter

1. After presentation:

- Another research adviser asks control questions, max 2 min:
- (to audience) Did the problem become clear? Repeat questions from guidelines to presenter
- Same with solution, vision
- Even though some questions about the science may be necessary in order to understand the idea: No lengthy discussions about the science. Keep the discussion about the clarity of idea presentation.  Adviser (time keeper) will stop discussions going too much into scientific detail.
