# Supplementary material for: Proposal Writing Training and Idea Development for Early‐Career Researchers Based on Constructive Alignment, Co‐Creation and Active Learning Strategies
Source: Ecol Evol. 2025 Oct 27;15(10):e72162. doi: 10.1002/ece3.72162 (PMC12559024; doi:10.1002/ece3.72162)
Supplement: Supplementary file 3 — Appendix S3: Bjerknes workshop: Writing successful project proposals—2024. [file ECE3-15-e72162-s002.pptx]

## Slide 1
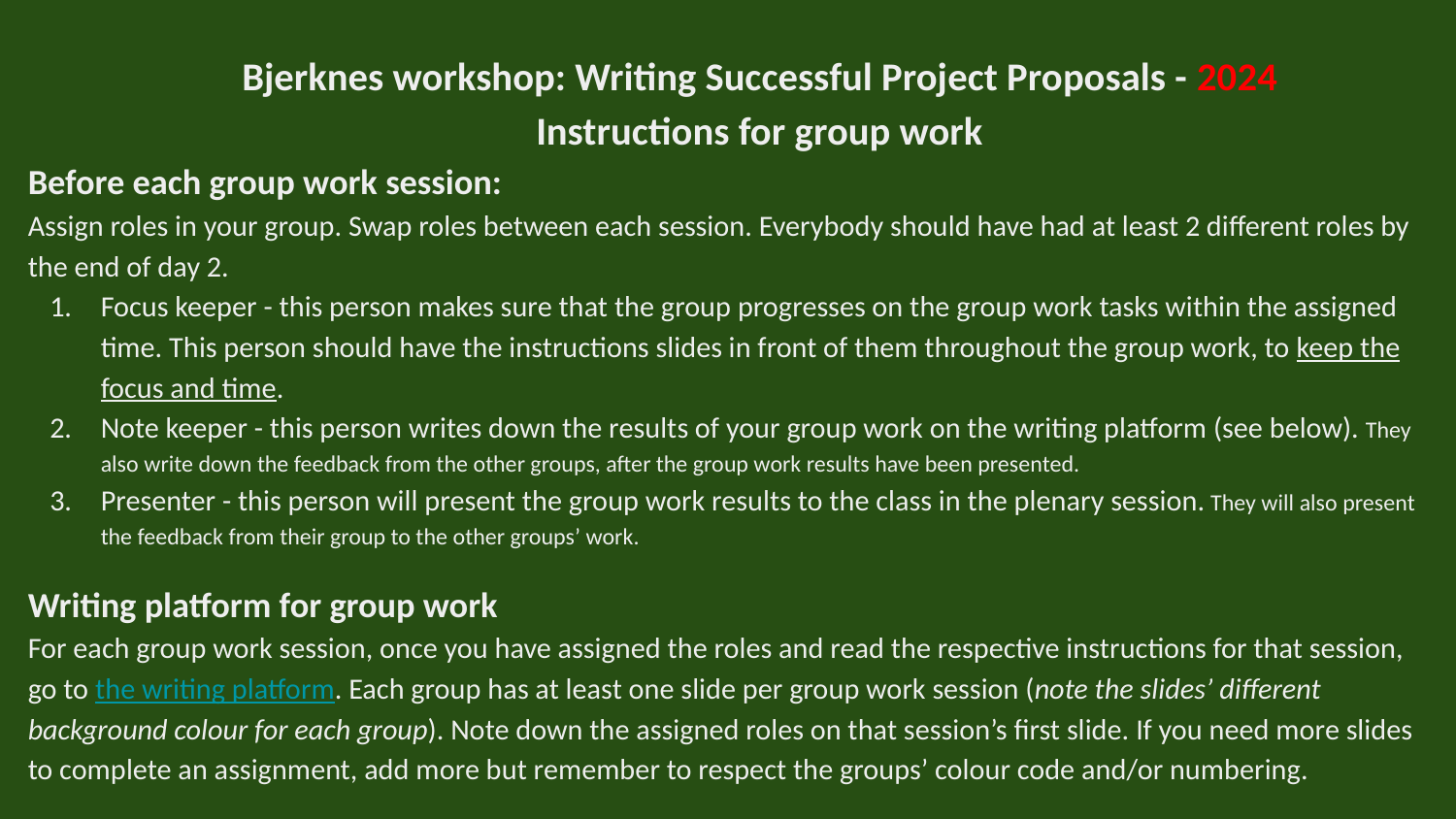

Bjerknes workshop: Writing Successful Project Proposals - 2024
Instructions for group work
Before each group work session:
Assign roles in your group. Swap roles between each session. Everybody should have had at least 2 different roles by the end of day 2.
Focus keeper - this person makes sure that the group progresses on the group work tasks within the assigned time. This person should have the instructions slides in front of them throughout the group work, to keep the focus and time.
Note keeper - this person writes down the results of your group work on the writing platform (see below). They also write down the feedback from the other groups, after the group work results have been presented.
Presenter - this person will present the group work results to the class in the plenary session. They will also present the feedback from their group to the other groups’ work.
Writing platform for group work
For each group work session, once you have assigned the roles and read the respective instructions for that session, go to the writing platform. Each group has at least one slide per group work session (note the slides’ different background colour for each group). Note down the assigned roles on that session’s first slide. If you need more slides to complete an assignment, add more but remember to respect the groups’ colour code and/or numbering.

## Slide 2
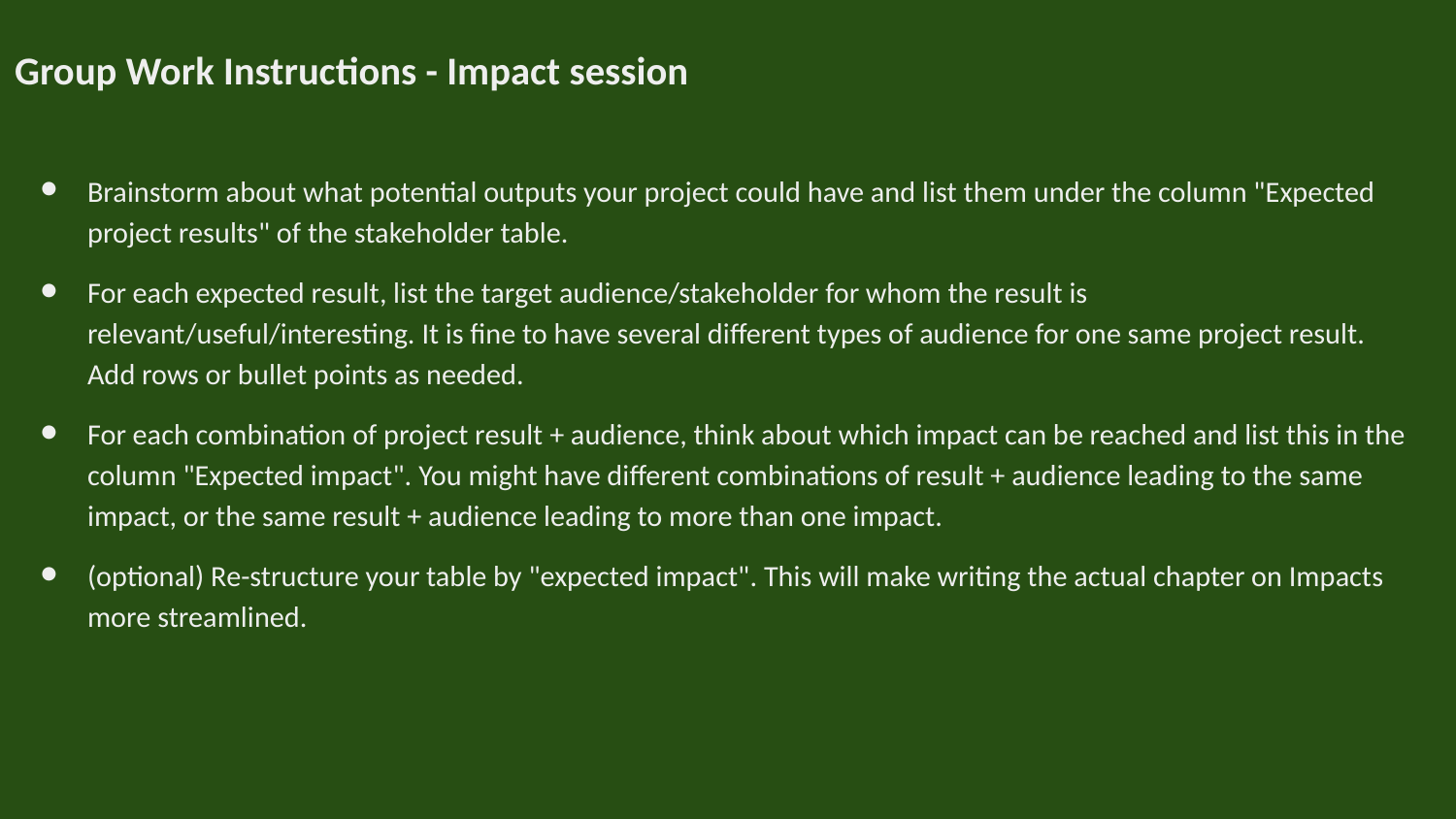

Group Work Instructions - Impact session
Brainstorm about what potential outputs your project could have and list them under the column "Expected project results" of the stakeholder table.
For each expected result, list the target audience/stakeholder for whom the result is relevant/useful/interesting. It is fine to have several different types of audience for one same project result. Add rows or bullet points as needed.
For each combination of project result + audience, think about which impact can be reached and list this in the column "Expected impact". You might have different combinations of result + audience leading to the same impact, or the same result + audience leading to more than one impact.
(optional) Re-structure your table by "expected impact". This will make writing the actual chapter on Impacts more streamlined.

## Slide 3
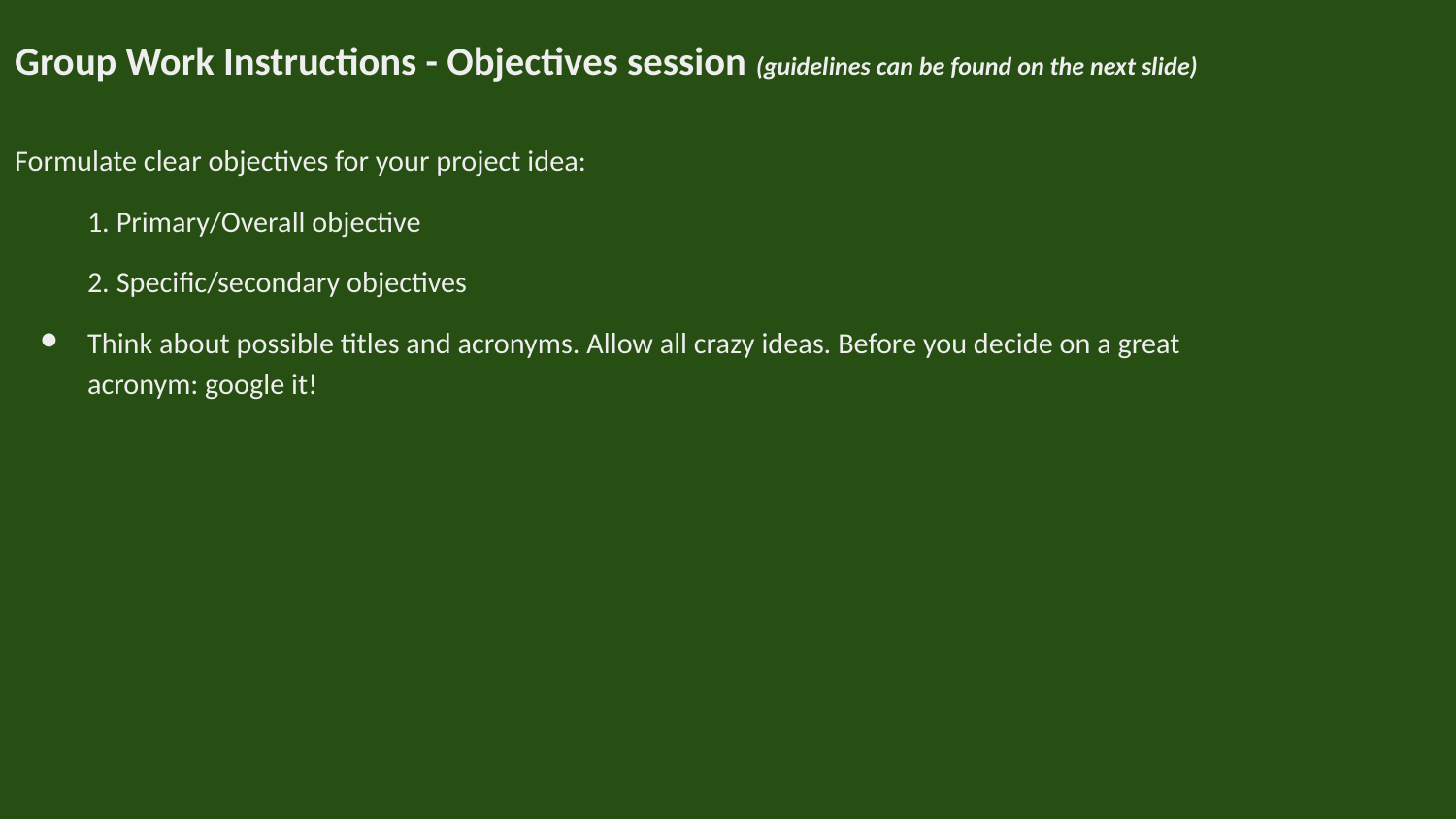

Group Work Instructions - Objectives session (guidelines can be found on the next slide)
Formulate clear objectives for your project idea:​
1. Primary/Overall objective​
2. Specific/secondary objectives
Think about possible titles and acronyms. Allow all crazy ideas. Before you decide on a great acronym: google it!

## Slide 4
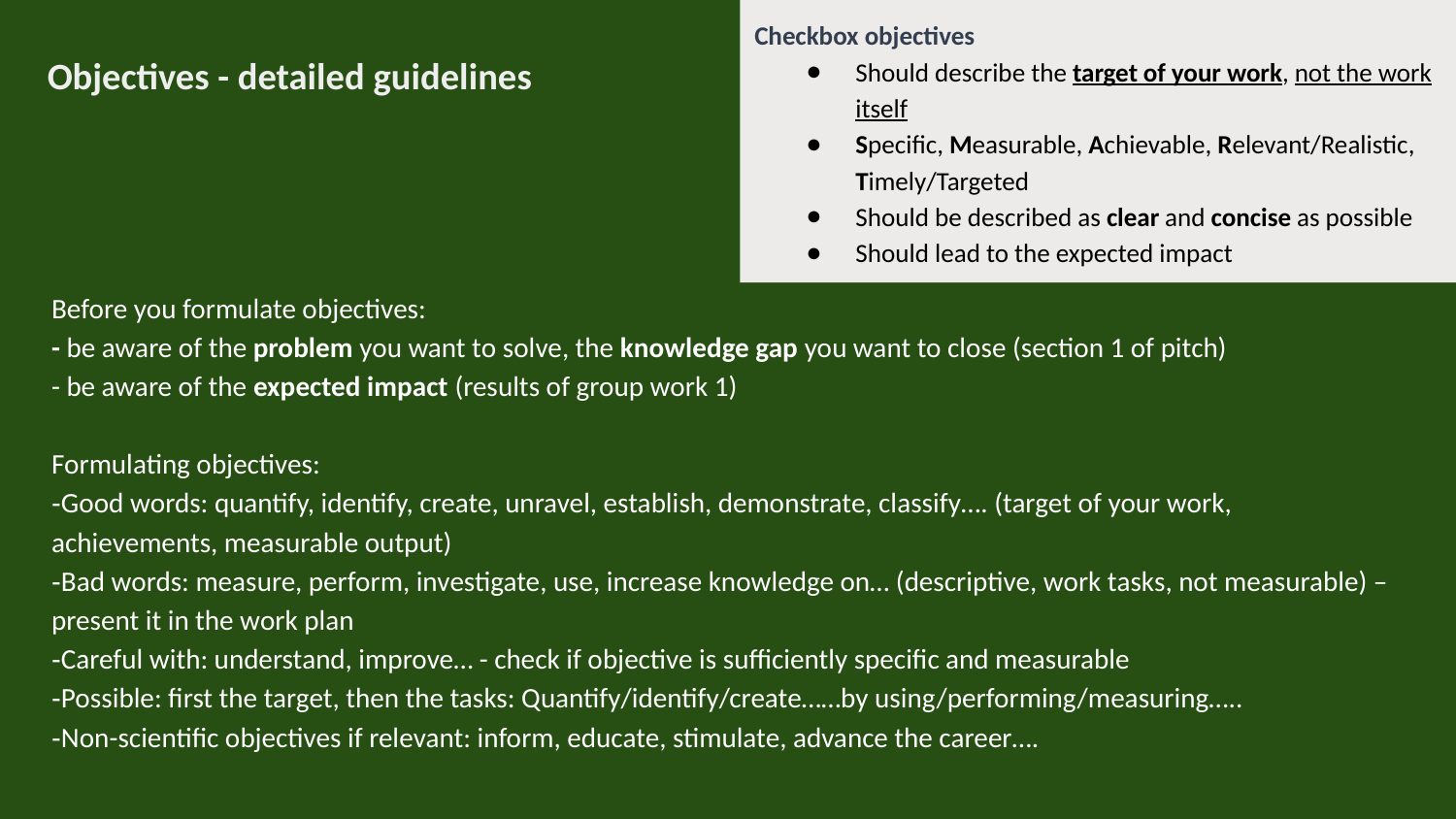

Checkbox objectives​
Should describe the target of your work, not the work itself​
Specific, Measurable, Achievable, Relevant/Realistic, Timely/Targeted​
Should be described as clear and concise as possible​
Should lead to the expected impact​
Objectives - detailed guidelines
Before you formulate objectives:
- be aware of the problem you want to solve, the knowledge gap you want to close (section 1 of pitch)
- be aware of the expected impact (results of group work 1)
Formulating objectives:
-Good words: quantify, identify, create, unravel, establish, demonstrate, classify…. (target of your work, achievements, measurable output)
-Bad words: measure, perform, investigate, use, increase knowledge on… (descriptive, work tasks, not measurable) – present it in the work plan
-Careful with: understand, improve… - check if objective is sufficiently specific and measurable
-Possible: first the target, then the tasks: Quantify/identify/create……by using/performing/measuring…..
-Non-scientific objectives if relevant: inform, educate, stimulate, advance the career….

## Slide 5
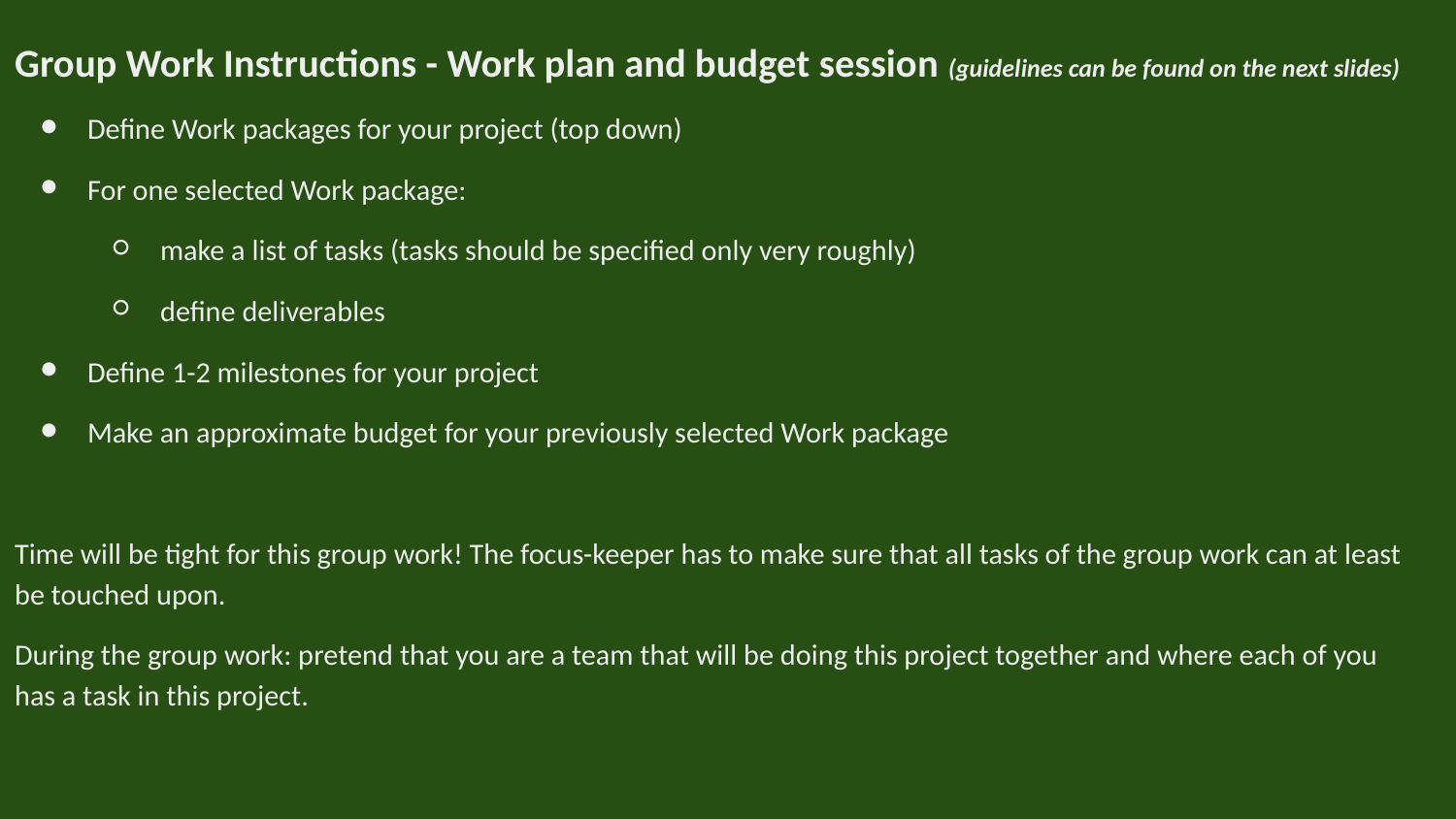

Group Work Instructions - Work plan and budget session (guidelines can be found on the next slides)
Define Work packages for your project (top down) ​
For one selected Work package:​
make a list of tasks (tasks should be specified only very roughly)​
define deliverables​
Define 1-2 milestones for your project​
Make an approximate budget for your previously selected Work package
Time will be tight for this group work! The focus-keeper has to make sure that all tasks of the group work can at least be touched upon. ​
During the group work: pretend that you are a team that will be doing this project together and where each of you has a task in this project.​

## Slide 6
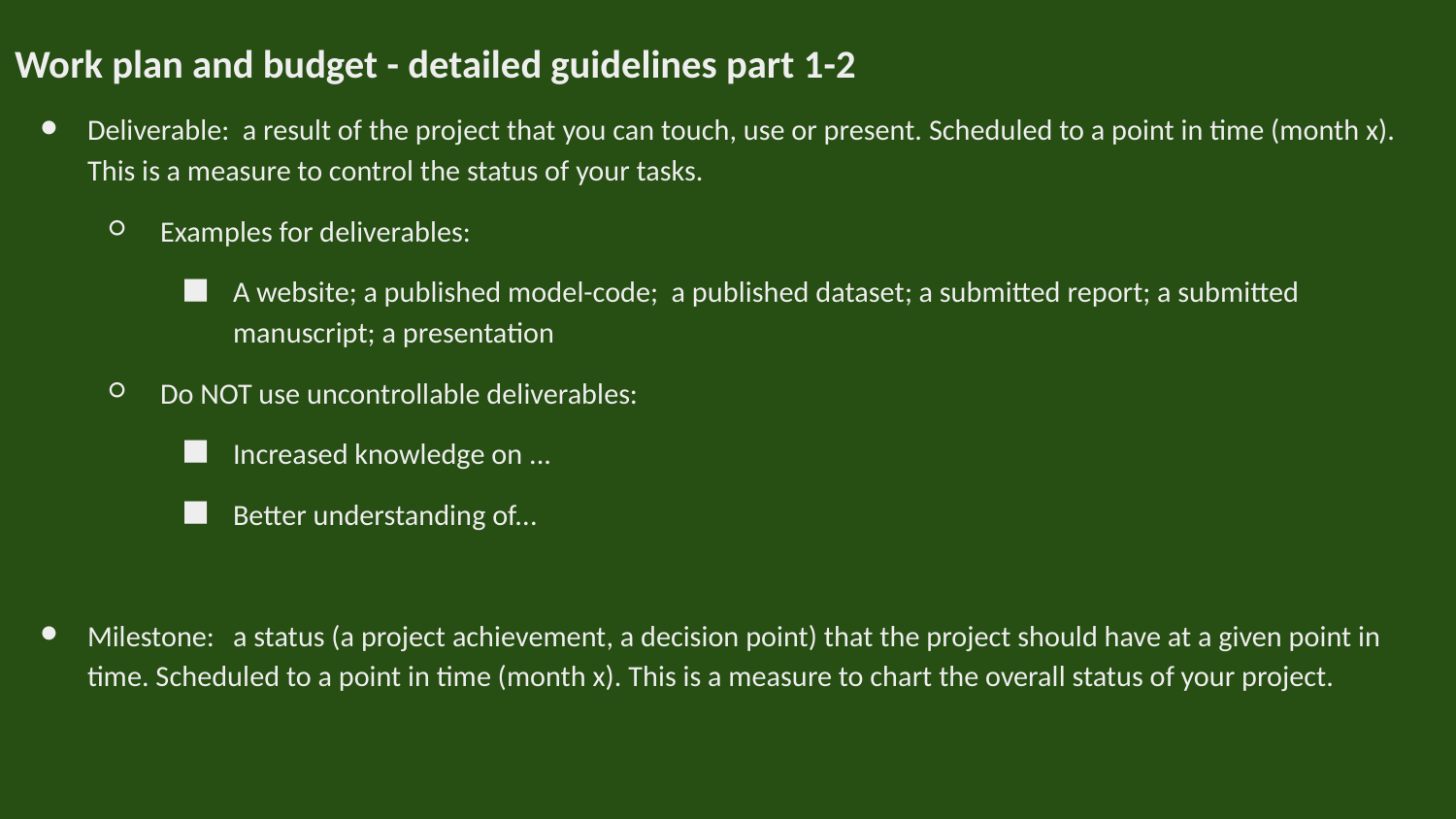

Work plan and budget - detailed guidelines part 1-2
Deliverable: a result of the project that you can touch, use or present. Scheduled to a point in time (month x). This is a measure to control the status of your tasks.
Examples for deliverables:​
A website​; a published model-code; a published dataset; a submitted report; a submitted manuscript; a presentation​
Do NOT use uncontrollable deliverables:​
Increased knowledge on ...​
Better understanding of...​
Milestone: 	a status (a project achievement, a decision point) that the project should have at a given point in time. Scheduled to a point in time (month x). This is a measure to chart the overall status of your project.

## Slide 7
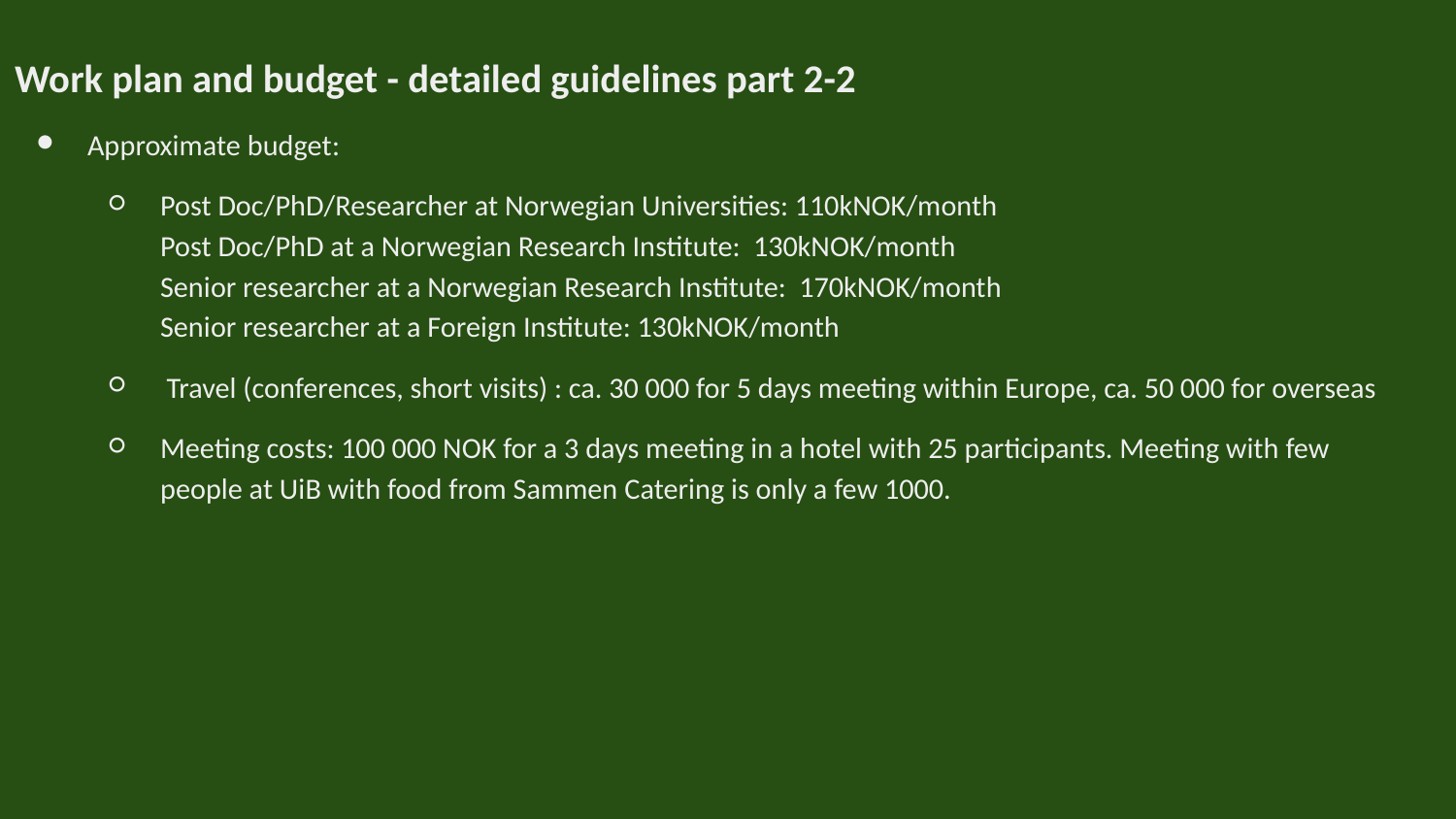

Work plan and budget - detailed guidelines part 2-2
Approximate budget:
Post Doc/PhD/Researcher at Norwegian Universities: 110kNOK/month
Post Doc/PhD at a Norwegian Research Institute: 130kNOK/month
Senior researcher at a Norwegian Research Institute: 170kNOK/month
Senior researcher at a Foreign Institute: 130kNOK/month
 Travel (conferences, short visits) ​: ca. 30 000 for 5 days meeting within Europe, ca. 50 000 for oversea​s
Meeting costs: ​100 000 NOK for a 3 days meeting in a hotel with 25 participants. Meeting with few people at UiB with food from Sammen Catering is only a few 1000.

## Slide 8
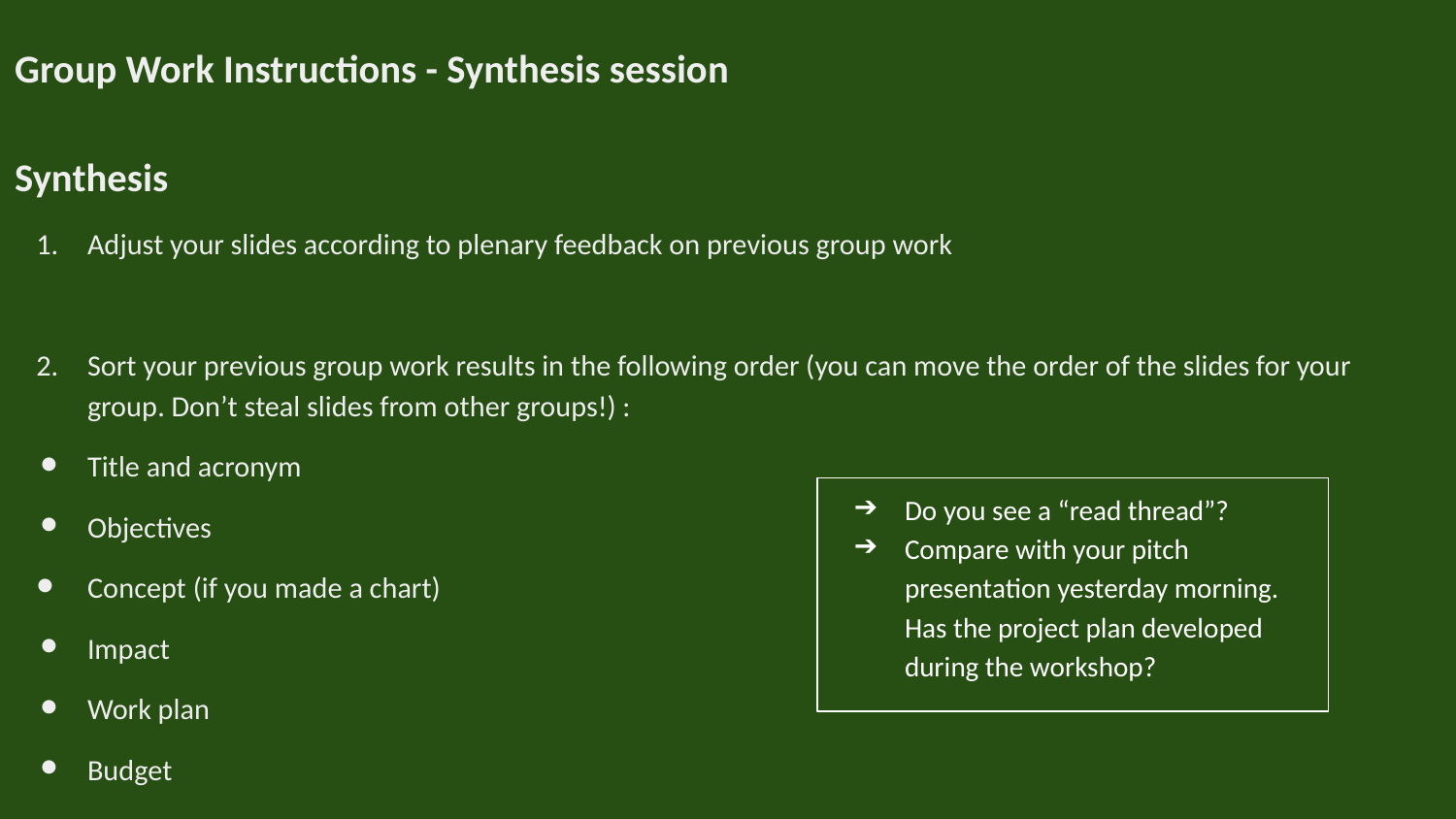

Group Work Instructions - Synthesis session
Synthesis
Adjust your slides according to plenary feedback on previous group work
Sort your previous group work results in the following order (you can move the order of the slides for your group. Don’t steal slides from other groups!) :
Title and acronym​
Objectives​
Concept (if you made a chart)
Impact​
Work plan
Budget
Do you see a “read thread”?
Compare with your pitch presentation yesterday morning. Has the project plan developed during the workshop?

## Slide 9
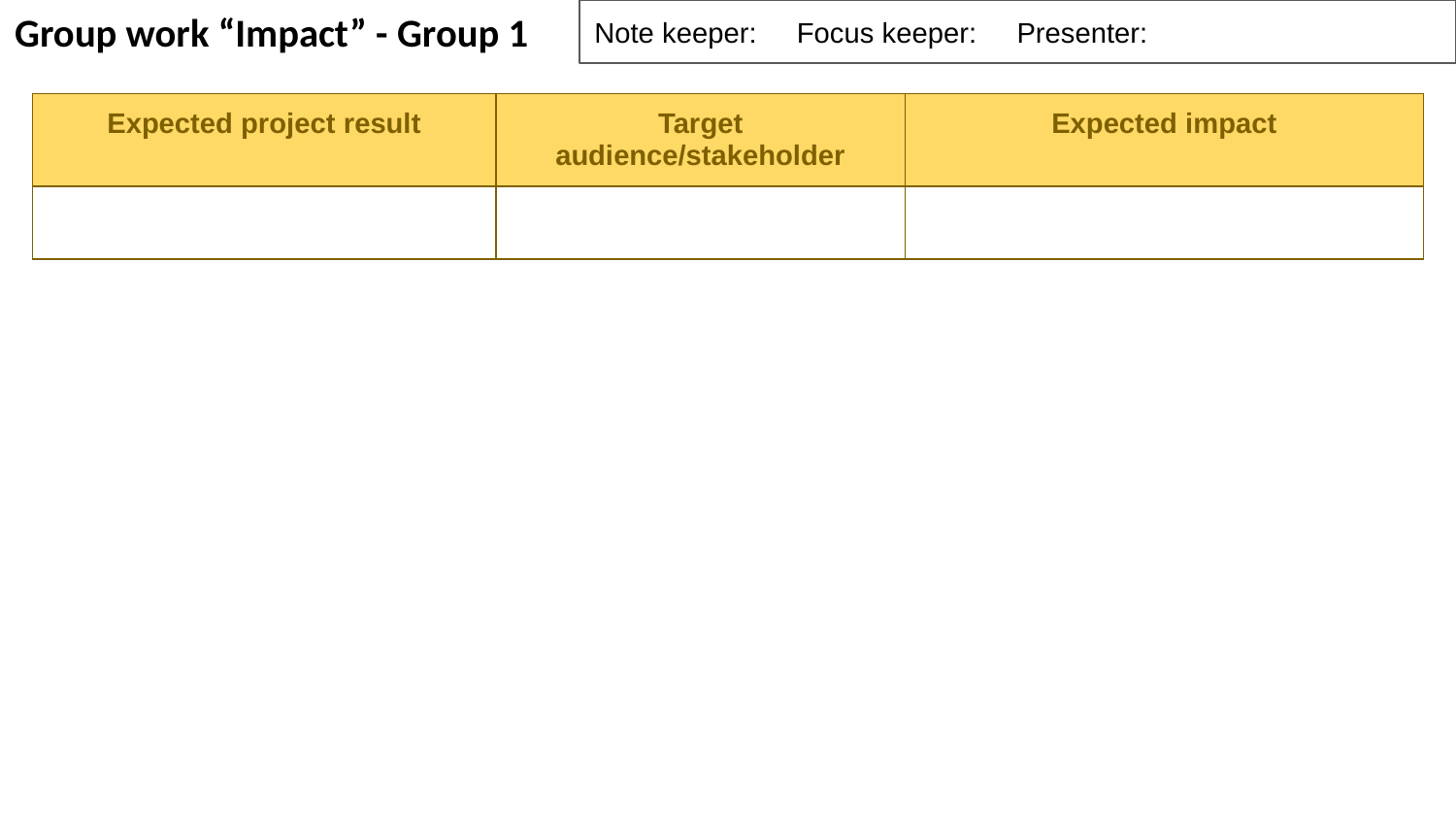

Note keeper: Focus keeper: Presenter:
Group work “Impact” - Group 1
| Expected project result | Target audience/stakeholder | Expected impact |
| --- | --- | --- |
| | | |

## Slide 10
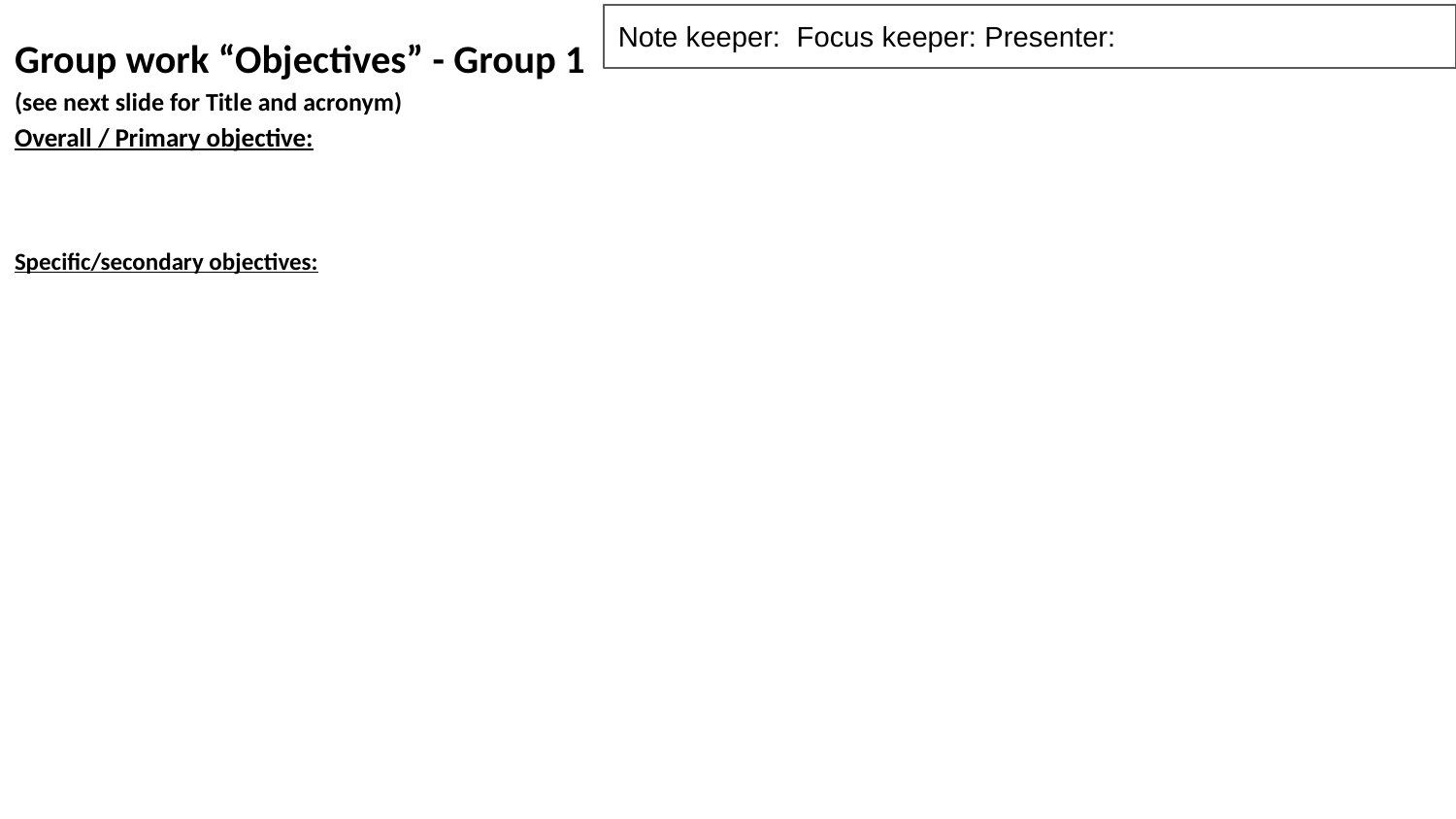

Group work “Objectives” - Group 1
(see next slide for Title and acronym)
Overall / Primary objective​:
Specific/secondary objectives:
Note keeper: Focus keeper: Presenter:

## Slide 11
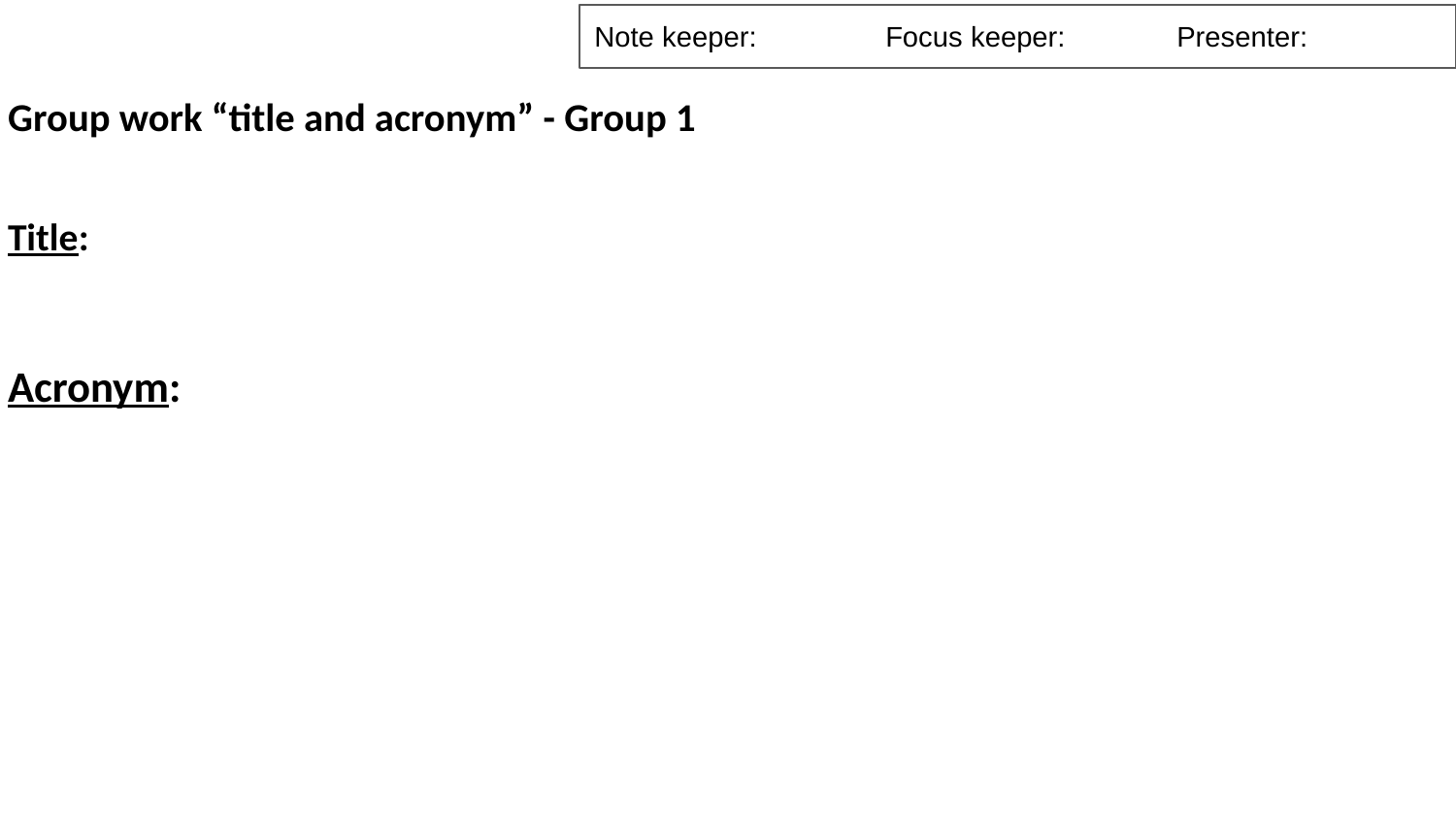

Group work “title and acronym” - Group 1
Title:
Acronym:
Note keeper:	Focus keeper:	Presenter:

## Slide 12
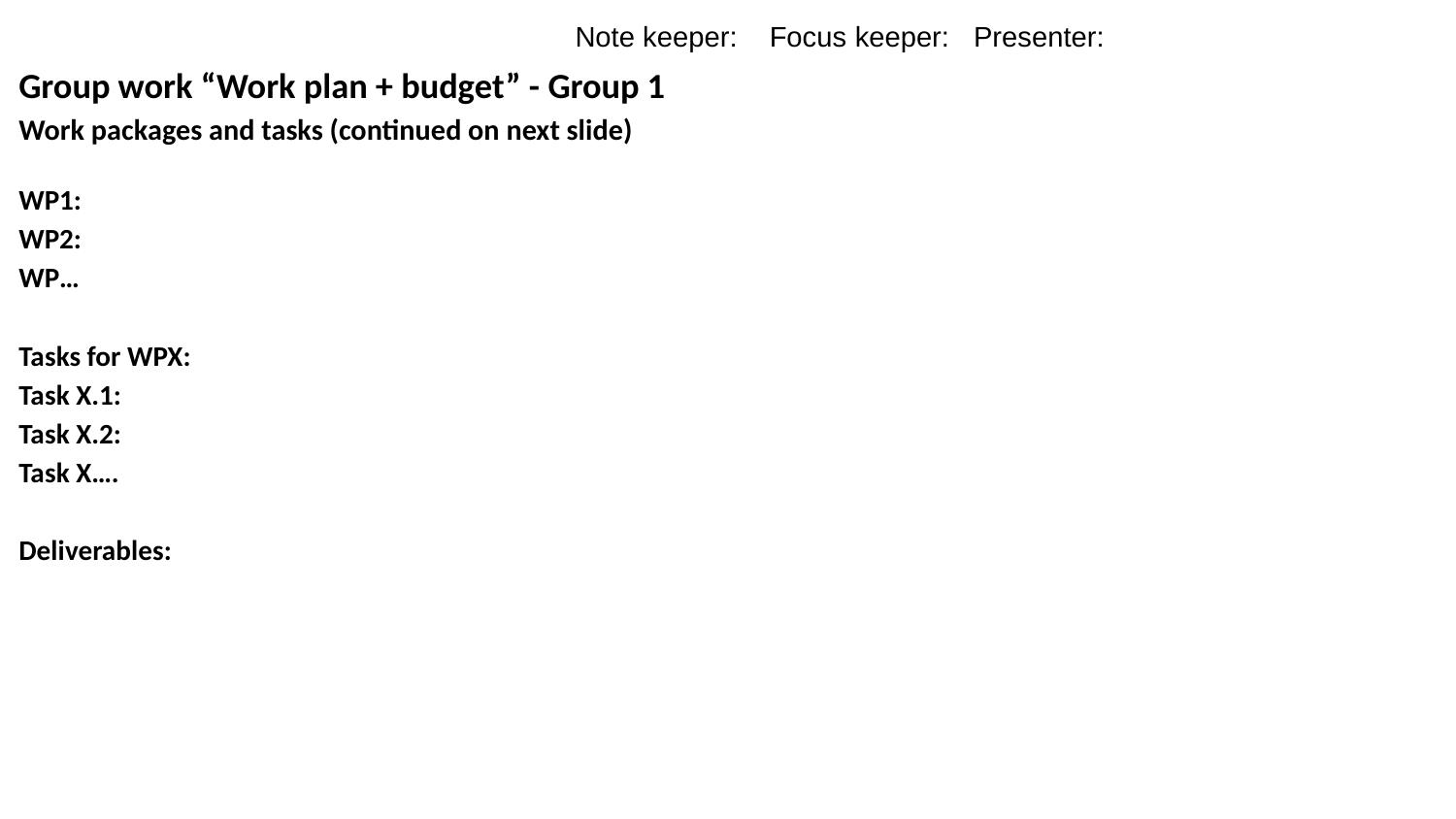

Group work “Work plan + budget” - Group 1
Work packages and tasks (continued on next slide)​
WP1:
WP2:
WP…
Tasks for WPX:
Task X.1:
Task X.2:
Task X….
Deliverables:
Note keeper: Focus keeper: Presenter:

## Slide 13
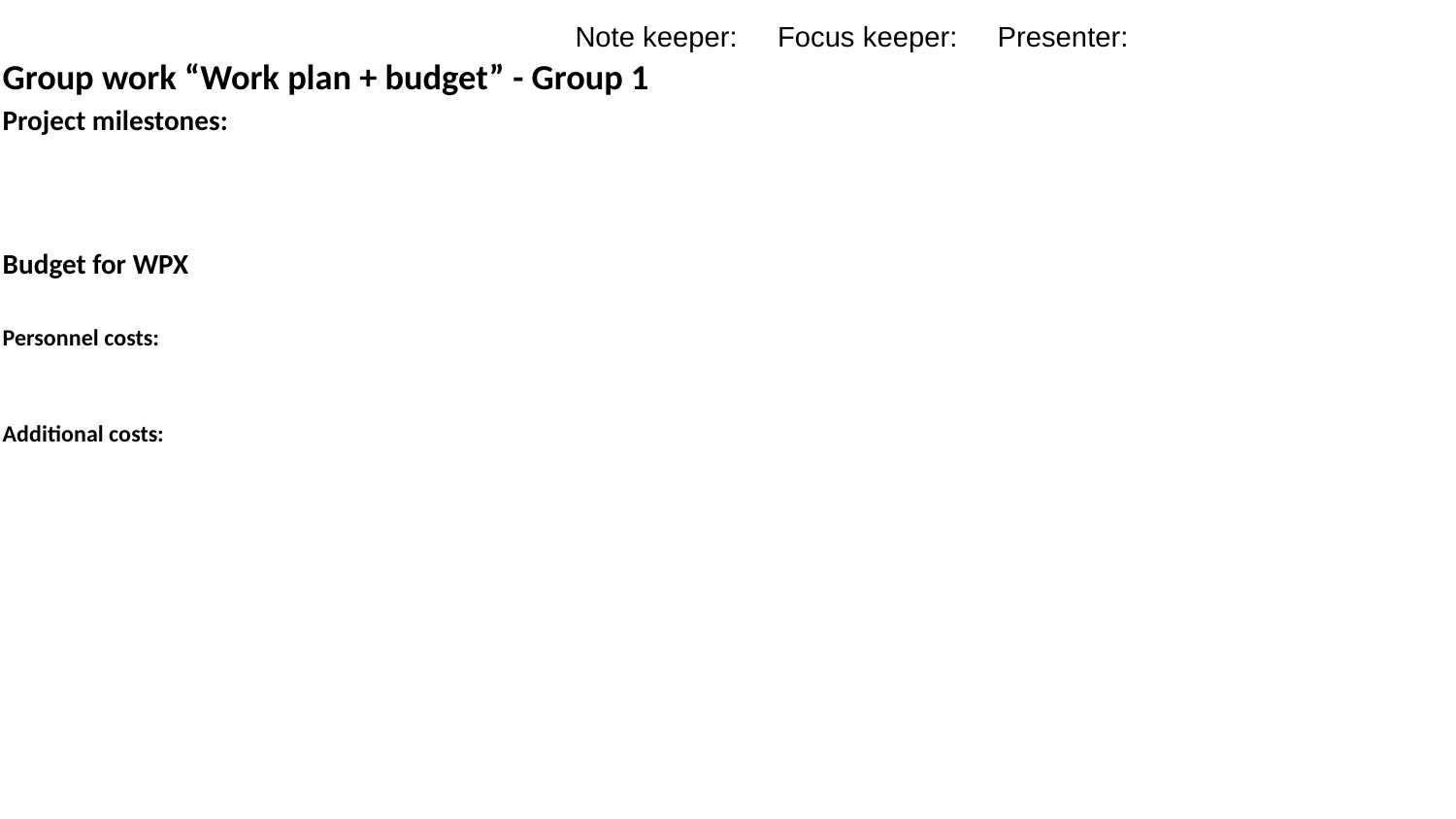

Group work “Work plan + budget” - Group 1
Project milestones:
Budget for WPX
Personnel costs:
Additional costs:​
Note keeper: Focus keeper: Presenter:

## Slide 14
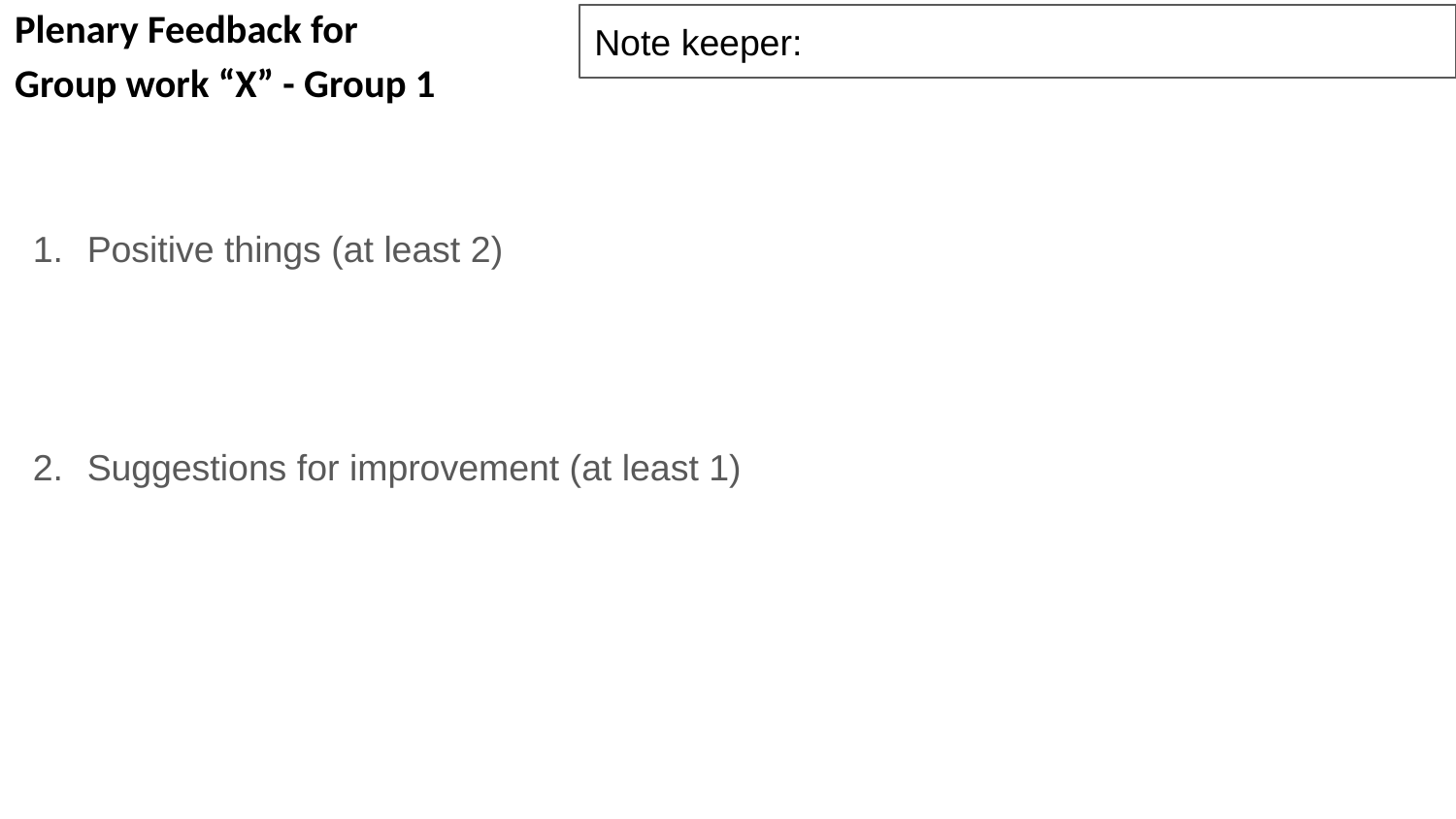

Plenary Feedback for
Group work “X” - Group 1
Note keeper:
Positive things (at least 2)
Suggestions for improvement (at least 1)
